# Highly Active and Stable Alkaline Hydrogen Evolution Electrocatalyst Based on Ir‐Incorporated Partially Oxidized Ru Aerogel under Industrial‐Level Current Density

**DOI:** 10.1002/advs.202307061

**Published:** 2023-12-10

**Authors:** Su Yan, Xiaojie Chen, Weimo Li, Mengxiao Zhong, Jiaqi Xu, Meijiao Xu, Ce Wang, Nicola Pinna, Xiaofeng Lu

**Affiliations:** ^1^ Alan G. MacDiarmid Institute, College of Chemistry Jilin University Changchun 130012 P. R. China; ^2^ Department of Chemistry, IRIS Adlershof and the Center for the Science of Materials Berlin Humboldt‐Universität zu Berlin Brook‐Taylor‐Straße 2 12489 Berlin Germany

**Keywords:** exceptional long‐term stability, hydrogen evolution reaction, industrial‐level current density, iridium‐incorporation, metal aerogel

## Abstract

The realization of large‐scale industrial application of alkaline water electrolysis for hydrogen generation is severely hampered by the cost of electricity. Therefore, it is currently necessary to synthesize highly efficient electrocatalysts with excellent stability and low overpotential under an industrial‐level current density. Herein, Ir‐incorporated in partially oxidized Ru aerogel has been designed and synthesized via a simple in situ reduction strategy and subsequent oxidation process. The electrochemical measurements demonstrate that the optimized Ru_98_Ir_2_‐350 electrocatalyst exhibits outstanding hydrogen evolution reaction (HER) performance in an alkaline environment (1 M KOH). Especially, at the large current density of 1000 mA cm^−2^, the overpotential is as low as 121 mV, far exceeding the benchmark Pt/C catalyst. Moreover, the Ru_98_Ir_2_‐350 catalyst also displays excellent stability over 1500 h at 1000 mA cm^−2^, denoting its industrial applicability. This work provides an efficient route for developing highly active and ultra‐stable electrocatalysts for hydrogen generation under industrial‐level current density.

## Graphical abstract

A facile strategy based on an in situ reduction and subsequent controllable oxidation process has been demonstrated to prepare Ir‐incorporated in partially oxidized Ru aerogel (Ru_98_Ir_2_‐350) with a 3D porous structure, which exhibits a remarkable HER performance in an alkaline solution. The as‐prepared Ru_98_Ir_2_‐350 catalyst shows highly active and ultra‐stable catalytic properties under industrial‐level current density, significantly superior to the commercial Pt/C and many previously reported catalysts.

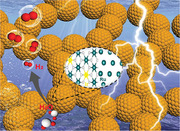

## 1 Introduction

Owing to the global energy and environmental crisis caused by the increasingly severe excessive consumption of traditional fossil fuels, renewable and clean substitutes are pressingly needed.^[^ ^1^, ^2^, ^3^, ^4^, ^5^ ^]^ Directly generating H_2_ through water electrolysis is one of the most researched strategies to produce green hydrogen for large‐scale industrial applications.^[^ ^6^, ^7^, ^8^, ^9^, ^10^ ^]^ To date, precious metal‐based material (e.g., Pt/C) is still regarded as the most active hydrogen evolution reaction (HER) electrocatalyst for water electrolysis in acidic medium.^[^ ^11^, ^12^, ^13^ ^]^ However, the state‐of‐the‐art Pt/C suffers durability issues at high current density. Although a considerable number of transition metal‐based HER electrocatalysts are designed and prepared, they are far from the expectations to achieve the standard of industrial application (current density up to 1000 mA cm^−2^) owing to their slow water activation kinetics caused by their active electronic states near the Fermi‐level. Therefore, exploiting highly active and stable electrocatalysts for the HER catalytic process at high current density is highly desirable but remains a challenge.

Recently, 3D metal aerogel materials have emerged as highly efficient and long‐lasting electrocatalysts for hydrogen production through water electrolysis due to their large specific surface area, porosity, large amount of active catalytic sites, outstanding conductivity to facilitate proton and electron transfer processes, and their self‐supporting architecture to ensure stability.^[^ ^14^, ^15^, ^16^, ^17^, ^18^ ^]^ For instance, Alexander and co‐workers initially investigate the influence of different polymetallic components of noble metal‐based aerogels toward HER.^[^ ^19^ ^]^ The resulting bimetallic Au‐Rh alloy aerogel shows outstanding HER activities under pH‐universal conditions and also surpasses the benchmark Pt/C catalyst. Interestingly, boron ‐doped osmium aerogel has also been synthesized via a simple and effective NaBH_4_ reduction method toward HER.^[^ ^20^ ^]^ Benefiting from the large porosity, numerous catalytic active sites, and the B doping to optimize the electronic structure and stabilize Os as active sites in an electron‐deficient state under realistic working conditions, the resultant B‐Os aerogel catalyst delivers better HER performances than Pt/C in three different electrolytes (acidic, alkaline, and neutral conditions). Recently, our group has designed a heterostructure consisting of Ru/RuO_2_ metal aerogel by partially oxidizing the pre‐fabricated single Ru metal aerogel.^[^ ^21^ ^]^ To produce a current density of 10 mA cm^−2^, the optimized Ru‐30 sample requires overpotentials of only 36 and 24 mV in alkaline and acidic media, respectively. Although those studies present exceptional HER activity, their long‐term stability is usually not satisfactory. Specifically, up to now, there is still no report on metal aerogel‐based HER electrocatalyst with a long‐term stability over 500 h at an industrial current density of 1000 mA cm^−2^.

Metal‐doping engineering has proven to be an effective strategy to promote the electrocatalytic activity and/or stability of catalysts for several electrochemical reactions by modifying their electronic structure to modulate the adsorption free energy of the intermediates and improve the electron transfer property.^[^ ^22^, ^23^ ^]^ As a typical example, nanosheets made of Fe‐doped Ni_5_P_4_ and Fe‐doped Ni(OH)_2_ have been fabricated, they deliver excellent oxygen evolution reaction (OER) and HER catalytic properties in alkaline media.^[^ ^24^ ^]^ Notably, recent studies have shown that the introduction of some transition metals (Mn, Ni, Nb, Rh) into RuO_2_ can significantly improve the electrocatalytic activity and stability toward OER in acidic conditions by reducing the adsorption of oxygen intermediates on active Ru sites.^[^ ^25^, ^26^, ^27^, ^28^ ^]^ However, it is still challenging to develop highly active and ultra‐stable HER electrocatalyst based on the RuO_2_ nanomaterials under industrial‐level current density.

Herein, we show that the incorporation of a small amount of Ir into partially oxidized Ru aerogel improves the alkaline HER activity at an industrial‐level current density. Our material only requires an overpotential of 121 mV to achieve a current density of 1000 mA cm^−2^ in 1 M KOH, significantly lower than that of the commercial Pt/C catalyst. More importantly, the optimized Ru_98_Ir_2_‐350 catalyst also shows outstanding long‐term stability over 1500 h at 1000 mA cm^−2^, which is better to the pure Ru_100_‐350 sample, commercial Pt/C and most of previously reported HER catalysts. Our findings confirm that the incorporation of Ir in the catalyst plays a pivotal role in preventing further oxidation of Ru during the HER process, resulting in notably enhanced durability. Our study offers promising avenues for the development of advanced metal oxide‐based electrocatalysts for HER.

## 2 Results and Discussion

### 2.1 Materials Preparation and Structural Characterization

**Figure 1:**
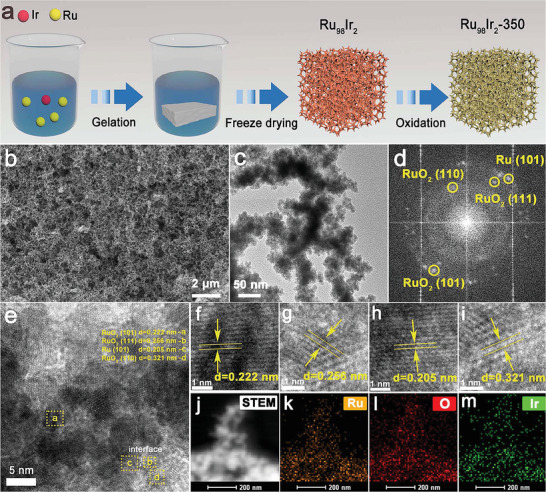
a) Schematic illustration of the synthesis procedure of Ru_98_Ir_2_‐350. b) FE‐SEM micrograph, c) TEM micrograph, d) FFT, e–i) HRTEM micrographs, j–m) HAADF‐STEM micrograph and corresponding EDX elemental mappings (at the Ru, O, and Ir edges) of Ru_98_Ir_2_‐350.

The synthesis of the Ir‐incorporated partially oxidized Ru metallic aerogel (Ru_98_Ir_2_‐350) can be divided into two steps. Initially, a freshly prepared sodium borohydride (NaBH_4_) aqueous solution is added to an aqueous solution of RuCl_3_ and IrCl_3_ with a molar ratio of 98:2 to produce Ru_98_Ir_2_ through an in situ co‐reduction route. Subsequently, the as‐prepared Ru_98_Ir_2_ intermediate product is oxidized at 350 °C to obtain Ir‐incorporated partially oxidized Ru aerogel (Ru_98_Ir_2_‐350), the detailed synthesis procedure is described in **Figure** **1**a. The other control samples with different molar ratios of Ru to Ir (Ru_100_, Ru_99_Ir_1_, Ru_95_Ir_5_, and Ru_90_Ir_10_) and their relevant oxidized products (Ru_100_‐350, Ru_99_Ir_1_‐350, Ru_95_Ir_5_‐350, and Ru_90_Ir_10_‐350) are synthesized via a similar procedure with that of Ru_98_Ir_2_ and Ru_98_Ir_2_‐350, respectively (detailed information are reported in the experimental section). The morphological properties of all the as‐prepared products are initially characterized by field‐emission scanning electron microscope (FE‐SEM). As presented in Figure S2 (Supporting Information), the as‐prepared Ru_98_Ir_2_ sample shows a conventional metal aerogel morphology composed of interconnected nanoparticles, and these nanoparticles randomly fuse at different angles to form a highly open and porous network.^[^ ^15^, ^29^ ^]^ After oxidation, the morphological characteristic of the corresponding Ru_98_Ir_2_‐350 is well‐retained compared to the original sample (Figure 1b). In addition, it can be also observed from the FE‐SEM images that all the other control samples including Ru_100_, Ru_99_Ir_1_, Ru_95_Ir_5_, Ru_90_Ir_10_ and their partially oxidized products possess a similar morphology to that of Ru_98_Ir_2_ and Ru_98_Ir_2_‐350 (Figure S3, Supporting Information). The transmission electron microscopy (TEM) image of Ru_98_Ir_2_‐350 further reveals that the constituent units of Ru_98_Ir_2_‐350 consist of branched aggregates (Figure 1c).

**Figure 2:**
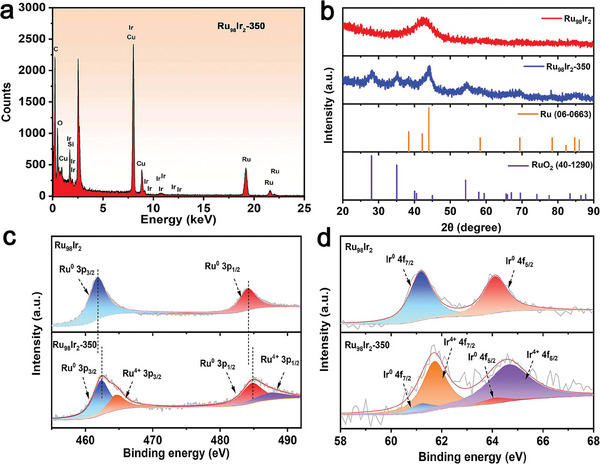
a) EDX spectrum of Ru_98_Ir_2_‐350. b) XRD profiles of Ru_98_Ir_2_ and Ru_98_Ir_2_‐350. c,d) Ru 3p and Ir 4f core XPS spectra of Ru_98_Ir_2_ and Ru_98_Ir_2_‐350.

From the high‐resolution transmission electron micrographs (HRTEM), four family of lattice fringes with interplanar spacings of 0.205, 0.222, 0.256, and 0.321 nm can be clearly observed in Figure 1e–i, which can be assigned to the (101) crystal plane of metallic Ru, (101) crystal plane of RuO_2_, (111) crystal plane of RuO_2_, and (110) crystal plane of RuO_2_, respectively. Additionally, their corresponding Debye‐Scherrer rings can be observed in the fast Fourier transform (FFT) patterns calculated from the Ru_98_Ir_2_‐350 HRTEM micrograph (Figure 1d). Through the analysis of HRTEM and FFT results, it is preliminarily confirmed that metallic Ru and RuO_2_ are present in the Ru_98_Ir_2_‐350 sample, further illustrating that a portion of metallic Ru is oxidized into RuO_2_ upon heat treatment. Owing to the extremely low content of Ir, no identified structural information of Ir species (Ir or IrO_2_) is found in the above characterizations. To further characterize the local composition and the presence of Ir, high‐angle annular dark‐field scanning (HAADF‐STEM) and relevant energy dispersive X‐Ray (EDX) elemental mapping are measured and shown in Figure 1j–m. Apart from Ru and O uniformly distributed in the aerogel structure, Ir can also be found in the EDX mapping of the Ru_98_Ir_2_‐350 sample. Moreover, the characteristic signals of Ru, Ir, and O elements are clearly observed in the EDX spectra further confirming the composition of the as‐prepared Ru_98_Ir_2_‐350 sample (**Figure** **2**a). Powder X‐ray diffraction (XRD) is also employed to shed light on the crystallographic structure of as‐prepared products. In the XRD patterns of the pre‐oxidized samples, only a broad peak at around 2θ = 43° appears, which can be indexed to the (101) plane of metallic Ru according to the standard PDF card No.06‐0663 (Figure 2b). After the heat treatment at 350 °C, a sharper XRD diffraction peak of 2θ = 43° and two additional peaks at 2θ = 38° and 42° appear, which correspond to the (100) and (002) planes of metallic Ru. Moreover, several low intensity peaks centered at 2θ = 28°, 35°, 40°, 54°, and 70° characteristic of RuO_2_ appear in the XRD pattern of Ru_98_Ir_2_‐350, which are assigned to the (110), (101), (111), (211), and (301) reflections of RuO_2_ (JCPDS Card No. 40–1290), respectively, further confirming the partial oxidation of Ru to RuO_2_. The other RuIr aerogels samples exhibit similar crystalline structures as the Ru_98_Ir_2_ and Ru_98_Ir_2_‐350 samples before and after annealing, respectively (Figure S4, Supporting Information).

X‐ray photoelectron spectroscopy (XPS) analysis is carried out to study the surface chemical composition and electronic properties of the samples. The survey spectrum of Ru_98_Ir_2_‐350 presents characteristic peaks of Ru, Ir, and O elements (Figure S5, Supporting Information), being consistent with the EDX results mentioned above. The Ru_98_Ir_2_ sample exhibits doublets at 461.8 and 484.1 eV in the high‐resolution Ru 3p spectrum, indexed to Ru^0^ 3p_3/2_ and Ru^0^ 3p_1/2_, respectively (Figure 2c).^[^ ^30^, ^31^ ^]^ The peaks located at 61.2 and 64.1 eV are attributed to Ir^0^ 4f_7/2_ and Ir^0^ 4f_5/2_, respectively (Figure 2d).^[^ ^8^, ^32^, ^33^ ^]^ The characteristic signatures of zero valent Ru and Ir indicate that Ru^3+^ and Ir^3+^ species from the metal salts are fully reduced to metallic species (Ru^0^ and Ir^0^) during the NaBH_4_ reduction procedure. It can be observed that the metallic Ru peaks of the Ru_98_Ir_2_‐350 sample after annealing exhibits shift to a higher binding energy value in comparison with that of Ru_98_Ir_2_, demonstrating electron transfer from Ru to RuO_2_ in the Ru/RuO_2_ heterointerface, which is consistent with previous reports.^[^ ^34^ ^]^ Additionally, two newly peaks centered at 464.6 and 487.3 eV appear in the Ru 3p core spectrum of Ru_98_Ir_2_‐350, ascribed to Ru^4+^ 3p_3/2_ and Ru^4+^ 3p_1/2_, respectively, further suggesting the partial oxidation of the sample.^[^ ^35^ ^]^ This result is well consistent with HRTEM and FFT results discussed above. In the Ir 4f core spectrum, in addition to the weak Ir^0^ peaks, a doublet at higher binding energies of 61.7 and 64.6 eV for Ru_98_Ir_2_‐350 sample is dominated, which are assigned to Ir^4+^ 4f_7/2_ and Ir^4+^ 4f_5/2_, respectively.^[^ ^36^ ^]^ The formation of high valence Ir may be due to the incorporation of Ir into the lattice of RuO_2_ during the annealing process. From the above structural and morphological characterizations, it can be affirmed that the as‐prepared Ru_98_Ir_2_‐350 product possesses a primary high valence state Ir‐incorporated into the Ru/RuO_2_ aerogel structure. Additionally, the Raman spectra of the as‐prepared samples are measured to further investigate the changes in metal valence states of materials after oxidation process. There are no obvious peaks appearing in the Raman spectrum of Ru_100_, whereas Ru_100_‐350 synthesized by subsequent oxidation process possesses two signal peaks located at 496 and 614 cm^−1^, which are indexed to Ru─O bond, demonstrating the generation of RuO_2_ (Figure S6, Supporting Information).^[^ ^37^ ^]^ The newly peaks related to RuO_2_ also appear in the Raman spectra of other products. It is worth noting that the two peaks indexed to RuO_2_ show a slight positive shift as the Ir content increases in the oxidized samples, which can be attributed to the successfully incorporation of Ir into RuO_2_ lattice to induce the lattice distortion and defects by nonstoichiometry according to previous literatures.^[^ ^38^ ^]^ Furthermore, two very weak peaks of Ir─O bonds located at around 558 and 725 cm^−1^ appear in the Raman spectrum of Ru_90_Ir_10_‐350, effectively indicating the presence of Ir^4+^ in the oxidized sample.^[^ ^39^ ^]^

### 2.2 Electrocatalytic HER Performance Evaluation

**Figure 3:**
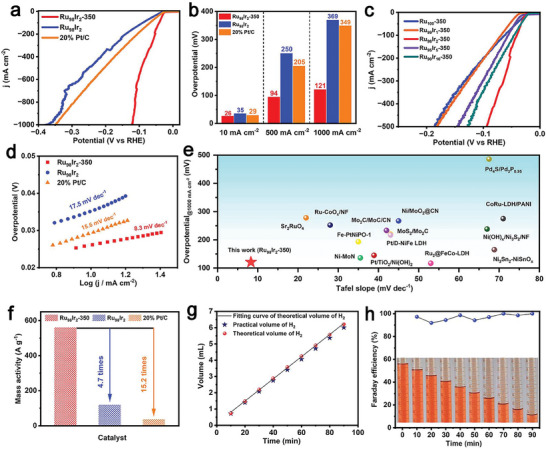
a) LSV curves of the as‐synthesized Ru_98_Ir_2_‐350, Ru_98_Ir_2_ samples, and commercial Pt/C catalyst. b) The overpotential comparison at the current density of 10, 500, and 1000 mA cm^−2^. c) LSV curves of the Ru_100_‐350, Ru_99_Ir_1_‐350, Ru_98_Ir_2_‐350, Ru_95_Ir_5_‐350, and Ru_90_Ir_10_‐350 samples. d) Tafel plots of the as‐synthesized Ru_98_Ir_2_‐350, Ru_98_Ir_2_ samples, and commercial Pt/C catalyst. e) Comparison of overpotential at 1000 mA cm^−2^ and Tafel slope value of the Ru_98_Ir_2_‐350 product with other highly active HER electrocatalysts. f) Mass activity value at an overpotential of 100 mV. g) The comparison of theoretically calculated and experimentally measured H_2_ evolution of Ru_98_Ir_2_‐350. h) Photos of recording changes in liquid level scale in Faraday testing.

The electrocatalytic behavior of all the as‐prepared products toward alkaline HER is evaluated in 1 M KOH electrolyte (pH = 14) with a conventionally three‐electrode electrochemical cell. In order to compare with commercial catalysts, the HER performance of Pt/C is also measured under the same conditions. **Figure** **3**a displays the linear sweep voltammetry (LSV) curves with a 100% *iR* compensation. It can be observed that the as‐prepared Ru_98_Ir_2_ sample exhibits slightly lower HER electrocatalytic property than the benchmark Pt/C material. Notably, the Ru_98_Ir_2_‐350 sample after annealing delivers a superior catalytic activity, especially at large current density. Remarkably, at current densities of 10, 500, and 1000 mA cm^−2^, the Ru_98_Ir_2_‐350 only requires overpotentials of 26, 94, and 121 mV, respectively, which are much lower than those of the Ru_98_Ir_2_ (35, 250, and 369 mV) and commercial Pt/C (29, 205, and 349 mV). The comparison of the overpotentials is illustrated in Figure 3b. We have also evaluated the HER performance of various Ru_x_Ir_y_‐350 samples, it is found that the Ru_98_Ir_2_‐350 exhibits the best activity in comparison with all the other samples (Figure 3c). Furthermore, compared to other Ru_x_Ir_y_ samples, their corresponding oxidized products display a significantly enhanced HER electrocatalytic activity (Figure S7, Supporting Information). Part of the reason for the improved HER performance can be attributed to the generation of the Ru/RuO_2_ heterostructure, which is beneficial to reducing hydrogen adsorption‐free energy (Δ*G*_H*_) according to our previous work.^[^ ^21^ ^]^ It is well known that RuO_2_ is inactive towards H* adsorption, while metallic Ru is active, thus the formation of Ru/RuO_2_ can modulate the H* adsorption and H_2_ desorption at its surface to optimize the HER activity. Herein, in our Ru_98_Ir_2_‐350 system, the incorporation of Ir into the Ru/RuO_2_ heterostructure can reduce the oxidation of metallic Ru into RuO_2_ to maintain the Ru/RuO_2_ interface, thus improving the HER activity compared to pure Ru‐containing samples. This assumption is supported by the XPS results, which have been discussed in the latter section (Figure 5c,d). The remarkable HER electrocatalytic activity of Ru_98_Ir_2_‐350 is further supported by the Tafel plots derived from the LSV curves. As shown in Figure 3d and Figure S8 (Supporting Information), Ru_98_Ir_2_‐350 presents an ultra‐low Tafel slope of 8.3 mV dec^−1^, which is better than the commercial Pt/C catalyst (15.6 mV dec^−1^) and other as‐prepared samples (9.8–31.8 mV dec^−1^). Figure 3e and Table S1 (Supporting Information) present the comparison of the overpotential at 1000 mA cm^−2^ and Tafel slope value of the Ru_98_Ir_2_‐350 product with other previously reported highly active HER electrocatalysts. The comparison points to the superior HER performance of our Ru_98_Ir_2_‐350 sample, demonstrating the achievement of electrocatalytic H_2_ production at industrially‐compatible current densities.

The mass activity of various catalysts is further calculated to estimate their intrinsic activity. As displayed in Figure 3f, Ru_98_Ir_2_‐350 delivers a higher mass activity value of 560.7 A g^−1^ at an overpotential of 100 mV, which is 4.7 and 15.2 times as high as the one of Ru_98_Ir_2_ (119.4 A g^−1^) and Pt/C (36.8 A g^−1^), respectively. The price activity of Ru_98_Ir_2_‐350 also present at least 4.5 times as high as that of the benchmark Pt/C catalyst (Figure S9, Supporting Information), indicating the cost‐effectiveness of Ru_98_Ir_2_‐350. The charge transfer kinetics of all the catalysts are evaluated by electrochemical impedance spectroscopy (EIS). As depicted in Figure S10 (Supporting Information), the Ru_98_Ir_2_‐350 sample displays the smallest charge transfer resistance (R_ct_) value among all the samples. The double‐layer capacitance (C_dl_) extracted from cyclic voltammetry (CV) at different scan rates under non‐Faraday region is closely related to the electrochemical active surface area (ECSA) of the electrocatalyst (Figure S12, Supporting Information). As depicted in Figure S13 (Supporting Information), Ru_98_Ir_2_‐350 displays the largest C_dl_ value of 194.7 mF cm^−2^ in comparison to other samples (22.8–127.5 mF cm^−2^). According to the C_dl_ results, the ECSA values of all materials are further obtained based on the following equation: ECSA = C_dl_×S/C_s_, where C_s_ = 40 µF cm^−2^, the detailed calculation method is presented in the experimental section.^[^ ^40^, ^41^ ^]^ As listed in Table S1 (Supporting Information), Ru_98_Ir_2_‐350 presents the highest ECSA value of 954 cm^2^ among all the other samples. The Faraday efficiency (FE) of Ru_98_Ir_2_‐350 is also measured at 50 mA cm^−2^ with the water displacement method to further investigate the HER selectivity of the material. As shown in Figure 3g,h, the calculated FE value of Ru_98_Ir_2_‐350 within 90 min is ≈100%, indicating the high selectivity of Ru_98_Ir_2_‐350 toward H_2_ production.

**Figure 4:**
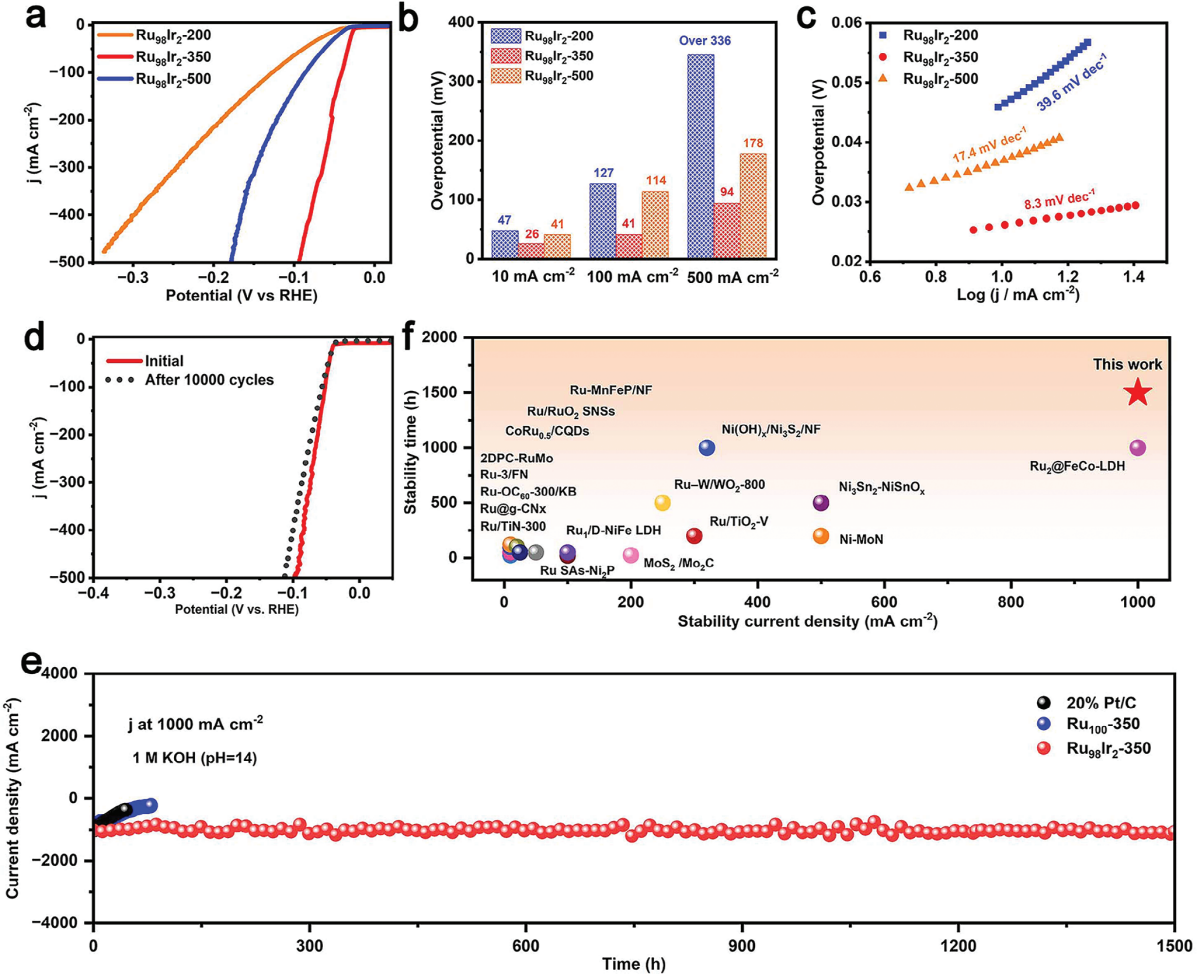
a) LSV curves of the Ru_98_Ir_2_‐200, Ru_98_Ir_2_‐350, and Ru_98_Ir_2_‐500 samples. b) Comparison of the overpotential at the current density of 10, 100, and 500 mA cm^−2^. c) Tafel plots of the Ru_98_Ir_2_‐200, Ru_98_Ir_2_‐350, and Ru_98_Ir_2_‐500 samples. d) LSV curves of Ru_98_Ir_2_‐350 before and after 10 000 CV cycles. e) i‐t continuous durability of Ru_98_Ir_2_‐350, Ru_100_, and commercial Pt/C at 1000 mA cm^‐2^. f) Comparison of the current density and time of the Ru_98_Ir_2_‐350 sample with literature reported HER electrocatalysts.

To estimate the influence of the oxidation temperature on the HER performance of Ru_98_Ir_2_, the Ru_98_Ir_2_‐200 and Ru_98_Ir_2_‐500 control samples are also fabricated through a similar synthesis procedure to that of Ru_98_Ir_2_‐350, but under different annealing temperature (200 and 500 °C, respectively). FE‐SEM images of Ru_98_Ir_2_‐200 and Ru_98_Ir_2_‐500 indicate similar morphologies compared to Ru_98_Ir_2_‐350, showing that the oxidation process does not have a significant impact on the morphology of the samples (Figure S14, Supporting Information). From the XRD pattern, it can be observed that as the annealing temperature increases, the XRD reflections assigned to RuO_2_ becomes stronger, revealing that more Ru in the aerogel is oxidized to RuO_2_ (Figure S15, Supporting Information). Ru_98_Ir_2_‐350 displays significantly lower overpotentials at 10, 100, and 500 mA cm^−2^ as well as lower Tafel slope values than those of Ru_98_Ir_2_‐500 and Ru_98_Ir_2_‐200. These results prove that the partially oxidized Ru_98_Ir_2_‐350 samples provides the best compromise leading to excellent HER electrocatalytic activity and reaction kinetics (**Figure** **4**a,b,c).

### 2.3 Electrocatalytic HER Stability Evaluation

The resistance of HER materials at a high current density is another important parameter for industrial H_2_ production applications. By comparing the LSV curves before and after 10 000 CV cycles (Figure 4d), it was found that the electrocatalytic activity of Ru_98_Ir_2_‐350 showed only a little decrease, demonstrating excellent CV stability. In addition, the i‐t curve at 1000 mA cm^−2^ is further used to estimate the long‐term HER stability of the catalyst at large current density. As shown in Figure 4e, after continuous i‐t testing for up to 1500 h, the current density of the catalyst only slightly decreases under constant applied potential. The stability of Ru_98_Ir_2_‐350 is much better than that of commercial Pt/C and mostly previous reported literatures (Figure 4f), confirming its superior durability at high current density. Notably, the Ru_98_Ir_2_‐350 sample also displays a greatly enhanced durability compared to the as‐prepared Ru_100_‐350 control sample. This result can be preliminarily attributed to the incorporation of Ir, which contributes in improving the stability of the Ru/RuO_2_ heterostructured material.

### 2.4 Characterization of Post‐HER Ru98Ir2‐350 and Mechanism of Ultra‐High Stability

**Figure 5:**
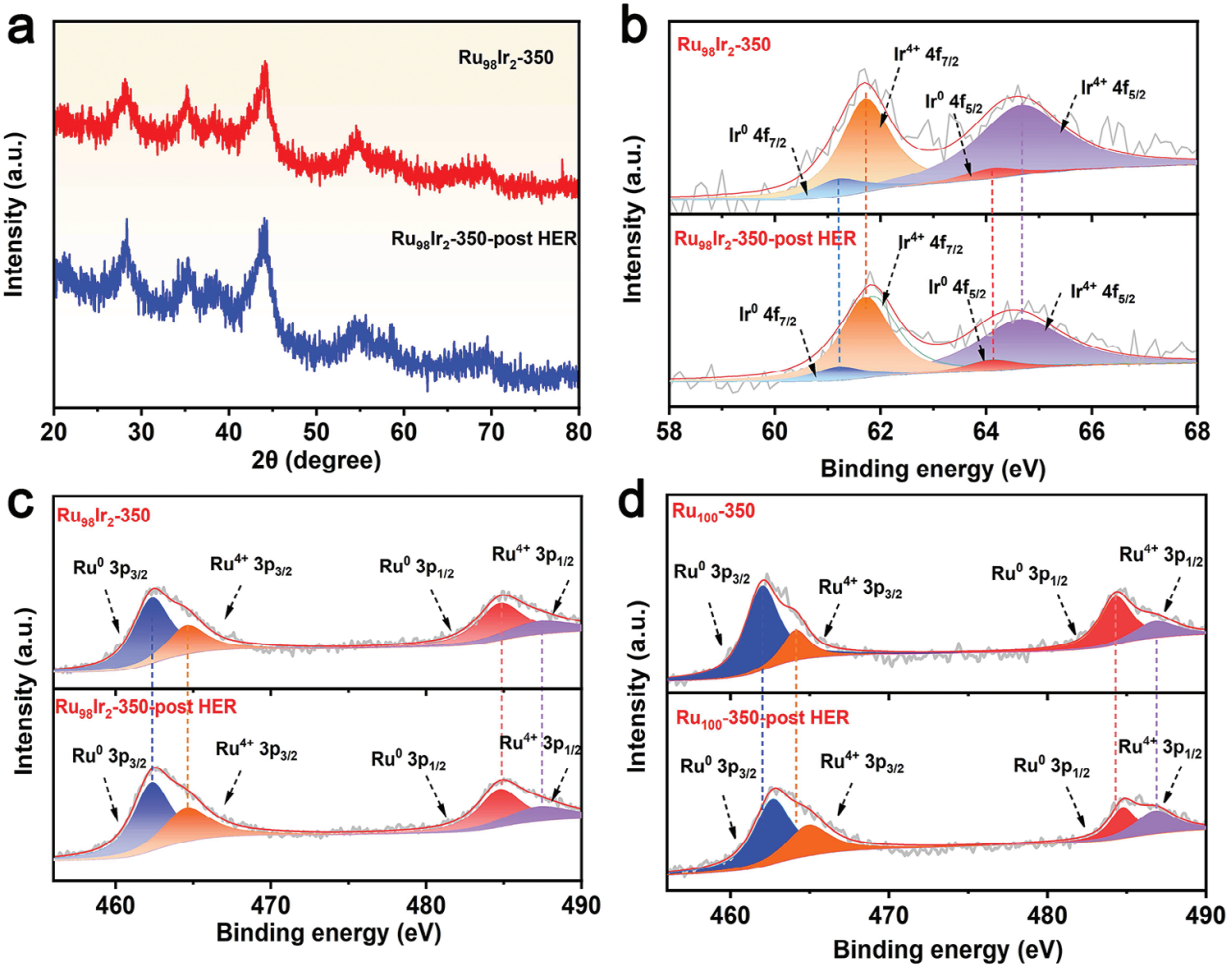
a) XRD patterns, b) Ir 4f XPS spectra, c) Ru 3p XPS spectra of Ru_98_Ir_2_‐350 before and after HER stability test in 1 M KOH. d) Ru 3p XPS spectra of the Ru_100_‐350 sample before and after HER durability measurement.

The morphological, compositional, and structural characterizations of Ru_98_Ir_2_‐350 after HER stability measurement in alkaline media is further estimated by FE‐SEM, XRD, and XPS. As presented in Figure S16 (Supporting Information), Ru_98_Ir_2_‐350 still exhibits an unchanged 3D porous morphology after long‐term operation. Moreover, all reflections in the powder XRD pattern exhibit no variation in comparison to the freshly prepared sample, indicating that the structure is not affected by the HER electrocatalytic process (**Figure** **5**a). The Ir 4f and Ru 3p XPS spectra of Ru_98_Ir_2_‐350 before and after HER durability measurement (Figure 5b,c) are also unchanged. The above results confirm that Ru_98_Ir_2_‐350 possesses an excellent structural and compositional stability during the HER electrocatalytic process. In order to better understand the stability of Ru_98_Ir_2_‐350, the Ru 3p XPS signal peaks of Ru_100_‐350 after the same durability test is investigated and compared. The binding energies corresponding to the Ru species (Ru^0^ and Ru^4+^) shows a positive shift to the higher binding energy values, illustrating that Ru species in the Ru_100_‐350 material (without Ir incorporation) are inclined to be oxidized to form a higher valence state during the HER process, which might be related to the etching of the Ru‐based materials by the alkaline electrolyte (Figure 5d). Whereas, the Ru oxidation state in Ru_98_Ir_2_‐350 is well maintained, thus the molar ratio of Ru and RuO_2_ in the catalyst remains constant, which results in an improvement of the long‐term stability. This result demonstrates that the introduction of Ir in the Ru/RuO_2_ heterostructure plays a key role in achieving an improvement of the long‐term stability.

## 3 Conclusion

In summary, we have demonstrated a systematic approach to design and fabricate a novel aerogel material consisting of Ir‐incorporated in partially oxidized Ru toward alkaline HER application. By combining the advantages of the unique 3D porous structure and the incorporation of Ir into Ru/RuO_2_ inhibits the further oxidation of metallic Ru during the HER process in an alkaline solution. The as‐derived optimized Ru_98_Ir_2_‐350 sample presents a remarkable electrocatalytic performance, requiring an ultralow overpotential of 121 mV to reach an industrial‐level current density of 1000 mA cm^−2^, accompanied by a low Tafel slope of 8.3 mV dec^−1^ and a high mass activity of 560.7 A g^−1^. These properties are significantly better than that of commercial Pt/C catalyst. More importantly, the Ru_98_Ir_2_‐350 also delivers an outstanding long‐term stability over 1500 h at 1000 mA cm^−2^, further reinforcing its potential for industrial‐scale hydrogen production applications. This study provides valuable insights for the development of HER electrocatalysts with both high activity and long‐term stability.

## 4 Experimental Section

### Chemicals and Materials

Iridium chloride (IrCl_3_·xH_2_O), ruthenium chloride (RuCl_3_·xH_2_O), and Nafion D‐117 dispersion were all purchased from Sigma‐Aldrich. Commercial Pt/C (20 wt%) material was provided by Johnson Matthey. Sodium borohydride (NaBH_4_) was acquired from Xilong Scientific Co. Ltd.

### Synthesis of RuIr Aerogel

In this work, five RuIr aerogel samples (Ru_100_, Ru_99_Ir_1_, Ru_98_Ir_2_, Ru_95_Ir_5_, and Ru_90_Ir_10_) with different Ru to Ir ratios were synthesized via a simple one‐step in situ NaBH_4_ reduction method. In a typical synthesis of Ru_98_Ir_2_, 0.098 mmol of RuCl_3_·xH_2_O and 0.002 mmol of IrCl_3_·xH_2_O (total metal salt was 0.1 mmol) were dissolved into 2 mL of deionized water in a 20 mL glass bottle, and sonicated for 1 min to form a homogenous solution. Next, the as‐fabricated fresh NaBH_4_ aqueous solution (0.3 mmol of NaBH_4_ dissolved in 3 mL of deionized water) was added to the above solution under 60 °C. After 60 min, a black precipitate was formed at the bottom of the flask. Afterward, the black precipitate was further washed with ultrapure water for 5 times to remove the residual impurities and then freeze‐dried overnight to obtain the Ru_98_Ir_2_ product. The synthesis of other various Ir‐doping Ru metal aerogels (Ru_100_, Ru_99_Ir_1_, Ru_95_Ir_5_, and Ru_90_Ir_10_) followed the same preparation procedure as that of the Ru_98_Ir_2_ sample with the appropriate stoichiometry.

### Synthesis of Ir‐Incorporated Partially Oxidized Ru Aerogel

The pre‐fabricated Ru_100_, Ru_99_Ir_1_, Ru_98_Ir_2_, Ru_95_Ir_5_, and Ru_90_Ir_10_ samples were further transferred into a muffle furnace and heated for 30 min at 350 °C to obtain Ir‐incorporated partially oxidized Ru aerogels, which were named as Ru_100_‐350, Ru_99_Ir_1_‐350, Ru_98_Ir_2_‐350, Ru_95_Ir_5_‐350, and Ru_90_Ir_10_‐350, respectively.

### Synthesis of Ru98Ir2‐200 and Ru98Ir2‐500

Similar to the procedure to prepare Ru_98_Ir_2_‐350, the pre‐fabricated Ru_98_Ir_2_ sample was transferred into a muffle furnace and heated at 200 and 500 °C for 30 min to obtain Ru_98_Ir_2_‐200 and Ru_98_Ir_2_‐500, respectively.

### Material Characterizations

The crystallographic structures of the as‐prepared samples were characterized by XRD (PANalytical B.V. Empyrean, Cu K_α_, λ = 1.5406 Å, 40 mA, and 40 kV). The morphological images of the as‐prepared samples were collected on the FE‐SEM (FEI Nova NanoSEM, 15 kV). The chemical valence state and surface electronic properties of the as‐synthesized samples were confirmed with XPS (Thermo Scientific ESCALAB250). The composition, element information, and atomic structural characterization of the samples were all acquired from HRTEM equipped with a EDX spectrometer (FEI Tecnai G2 F20). The electrochemical data of the samples were obtained from an electrochemical analyzer (CHENHUA, CHI660E). The Raman spectrum of as‐synthesized sample was tested by a LabRAM ARAMIS Smart Raman Spectrometer.

### Electrocatalytic Performance Measurements

The evaluation of the electrochemical properties of all the as‐prepared products toward HER in 1 M KOH electrolyte (pH = 14) were performed with a typical three‐electrode electrochemical cell at room temperature. Herein, catalyst‐coated carbon paper (loading area: 0.4×0.49 cm^2^), Hg/HgO electrode, and graphite rod were served as the working electrode, reference electrode, and counter electrode, respectively. The Hg/HgO electrode was calibrated before electrochemical tests as follows: two Pt plates polished with sandpapers were served as both the counter and working electrode, and then CV with two sweep segments was performed in the H_2_‐saturated 1 M KOH solution (1 mV s^−1^). The average value (−0.914 V) of the two potentials at the zero‐crossing point of the current was considered as the thermodynamic potential of the hydrogen electrode reaction, and the calibration result was illustrated in Figure S1 (Supporting Information). Therefore, the potential from Hg/HgO electrode can be converted to a reversible hydrogen electrode (RHE) using the following equation: E_RHE_ = E_Hg/HgO_ + 0.914 V. To fabricate the catalyst ink‐modified working electrode, 2 mg of the as‐synthesized electrocatalyst and 10 µL of Nafion D‐117 dispersion (5 wt%) were dispersed into 490 µL of a mixed solvent with a volume ratio of V_water_: V_isopropanol_ = 1:1. Then the above mixture was vigorously sonicated for 1 h to generate a homogeneous electrocatalyst ink. 50 µL of as‐prepared electrocatalyst ink (4 mg mL^−1^) was subsequently cast on the carbon paper electrode surface and dried in the air at room temperature, resulting in a mass loading density of 1 mg cm^−2^. For comparison, the working electrode fabrication procedure with the commercial 20% Pt/C catalyst was similar to that of the above samples and the corresponding mass loading density was 5 mg cm^−2^. All LSV curves of the samples were tested by scanning LSV with a scan rate of 1 mV s^−1^ and the CV curves for the estimation of the stability were measured with a scan rate of 100 mV s^−1^. All the LSV curves for all HER electrocatalytic measurements were obtained with a 100% of ohmic potential drop (iR) compensation. The EIS measurements were performed from 1–10^5^ Hz under an AC voltage of −0.98 V (vs Hg/HgO). The C_dl_ value of sample was obtained from CV measurements at various scan rates (50, 40, 30, 20, 10 mV s^−1^) under a non‐Faraday range of 0.19–0.29 V (vs RHE). The C_dl_ values were obtained by plotting the Δ*j*/2 (Δ*j* = *j*_a_ – *j*_c,_ where *j*_c_ and *j*_a_ correspond to the negative and positive current, respectively) at 0.24 V (vs RHE) versus scan rates. The ECSA value of sample was calculated from the equation: ECSA = C_dl_ × S/C_s_, where S was the geometric area of catalyst decorated‐electrode (0.196 cm^2^) and C_s_ was the value of the specific capacitance value (40 µF cm^−2^).^[^ ^40^, ^41^ ^]^ The mass activity values were evaluated with the equation: mass activity = j/m, where j was the current density (mA cm^−2^) at an overpotential of 100 mV and m was the mass loading of the catalysts. The applied voltage for i‐t stability test of Ru_98_Ir_2_‐350, Ru_100_‐350, and commercial Pt/C in 1 M KOH electrolyte was −1.62, −2.11, and −2.22 V (vs Hg/HgO), respectively. The FE calculation for H_2_ production was based on the following equation: FE = (z × F × n/I × t) × 100%, where z was 2 (number of electrons needed to generate one H_2_ molecule), F was the Faraday constant (96 485.3 C mol^−1^), n was the moles of gas evolved, and I was the constant current applied for t min. The drainage method was performed to measure the volume of the generated H_2_ during HER process. The change of the liquid volume in the pipette before and after the HER reaction was taken as the volume of the generated H_2_.

## Conflict of Interest

The authors declare no conflict of interest.

## Supporting information

Supporting Information

## Data Availability

The data that support the findings of this study are available from the corresponding author upon reasonable request.
