# Supplementary material for: Highly Active and Stable Alkaline Hydrogen Evolution Electrocatalyst Based on Ir‐Incorporated Partially Oxidized Ru Aerogel under Industrial‐Level Current Density
Source: Adv Sci (Weinh). 2023 Dec 10;11(7):2307061. doi: 10.1002/advs.202307061 (PMC10870084; doi:10.1002/advs.202307061)
Supplement: Supplementary file 1 — Supporting Information [file ADVS-11-2307061-s001.pdf]

ADVANCED SCIENCE
Open Access

Supporting Information

for *Adv. Sci.*, DOI 10.1002/advs.202307061

Highly Active and Stable Alkaline Hydrogen Evolution Electrocatalyst Based on Ir-Incorporated Partially Oxidized Ru Aerogel under Industrial-Level Current Density

*Su Yan, Xiaojie Chen, Weimo Li, Mengxiao Zhong, Jiaqi Xu, Meijiao Xu, Ce Wang, Nicola Pinna and Xiaofeng Lu**

Supporting Information

Highly Active and Stable Alkaline Hydrogen Evolution Electrocatalyst Based on Ir-incorporated Partially Oxidized Ru Aerogel under Industrial-level Current Density

Su Yan, Xiaojie Chen, Weimo Li, Mengxiao Zhong, Jiaqi Xu, Meijiao Xu, Ce Wang, Nicola Pinna, and Xiaofeng Lu*

S. Yan, X. J. Chen, W. M. Li, M. X. Zhong, J. Q. Xu, M. J. Xu, Prof. C. Wang, Prof. X. F. Lu

Alan G. MacDiarmid Institute

College of Chemistry

Jilin University

Changchun 130012, P. R. China

E-mail: xflu@jlu.edu.cn

Prof. N. Pinna

Department of Chemistry

IRIS Adlershof and the Center for the Science of Materials Berlin

Humboldt-Universität zu Berlin

Brook-Taylor-Straße 2, 12489 Berlin, Germany

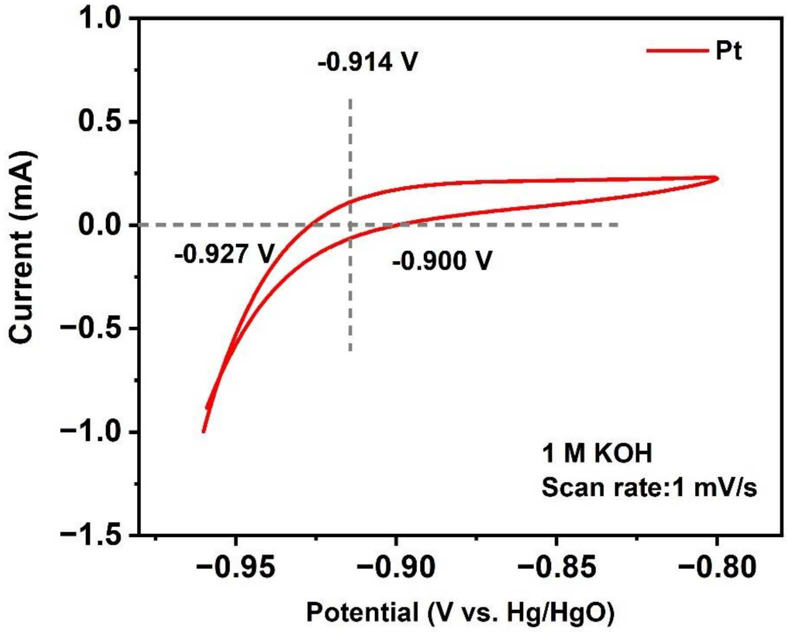

Figure S1. The calibration of Hg/HgO reference electrode.

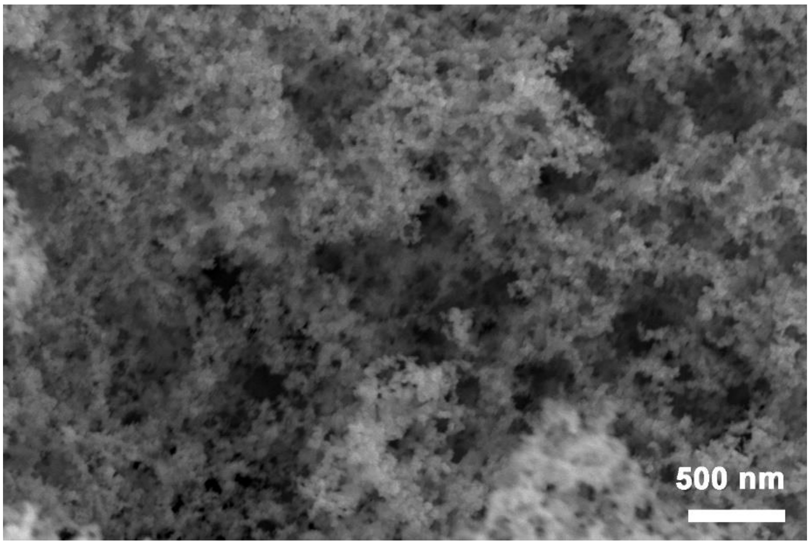

Figure S2. FE-SEM image of the as-synthesized $Ru_{98}Ir_2$ sample.

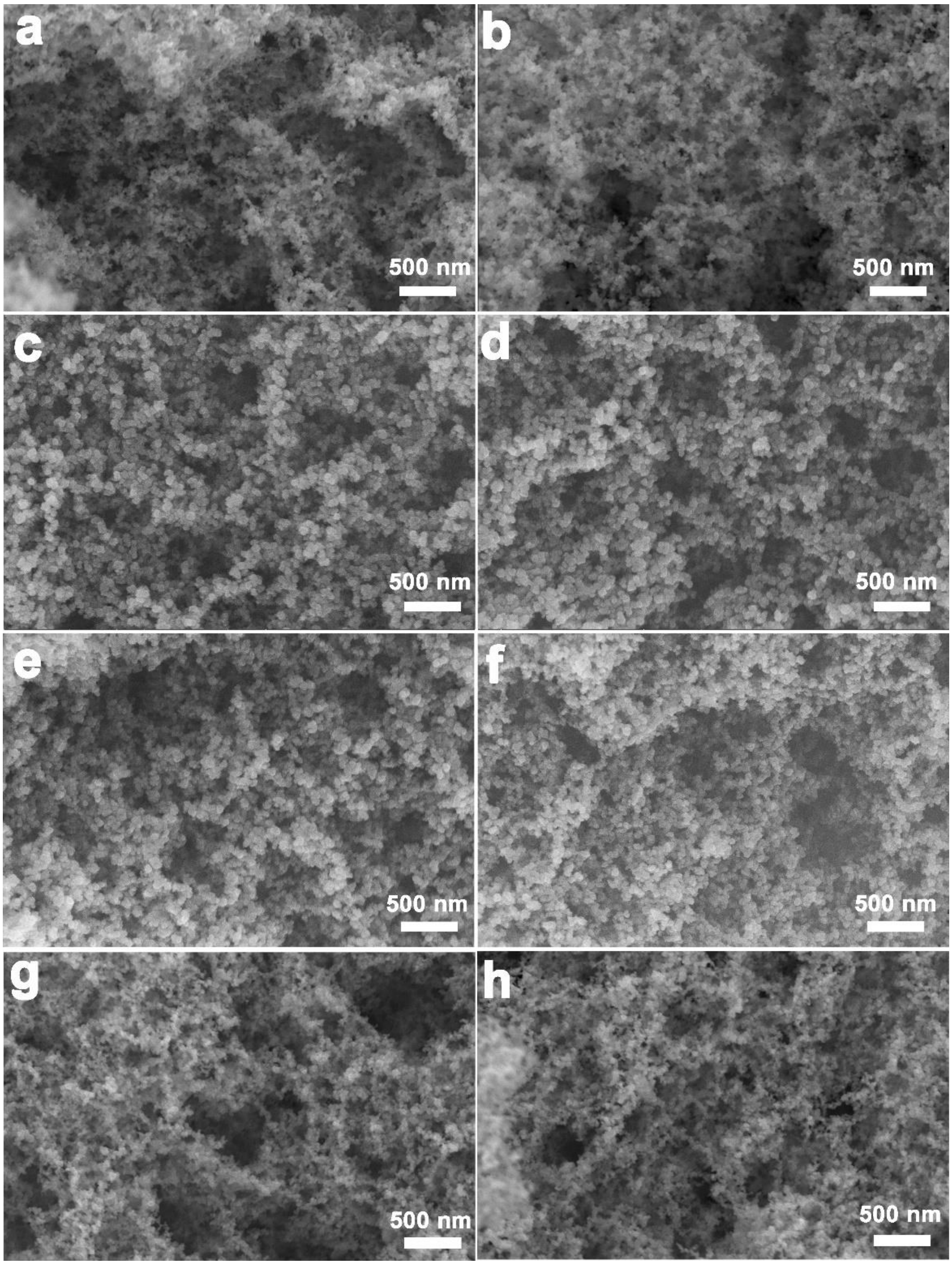

Figure S3. FE-SEM images of (a) Ru_{100}, (b) Ru_{100}-350, (c) $Ru_{99}Ir_1$, (d) $Ru_{99}Ir_1$-350, (e) $Ru_{95}Ir_5$, (f) $Ru_{95}Ir_5$-350, (g) $Ru_{90}Ir_{10}$, and (h) $Ru_{90}Ir_{10}$-350.

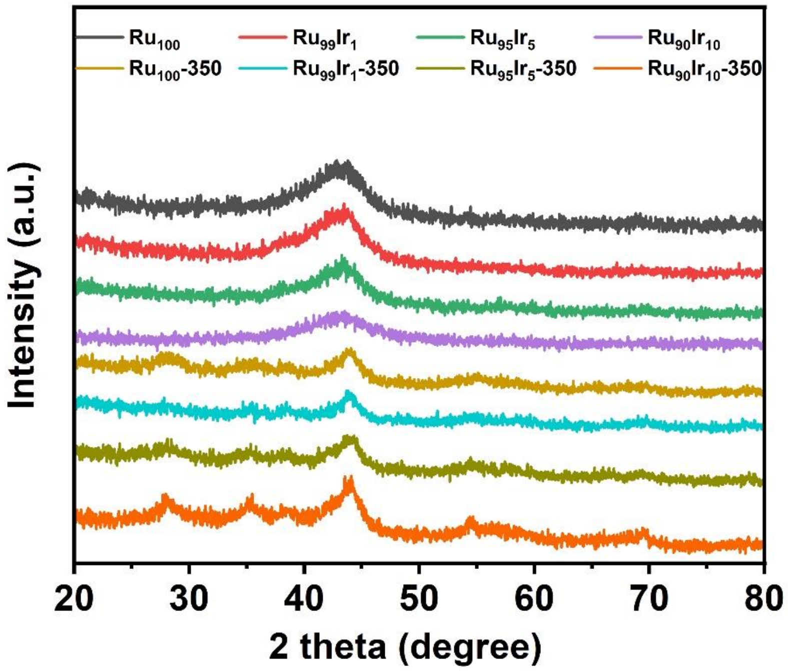

Figure S4. XRD patterns of Ru_{100}, Ru_{100}-350, $Ru_{99}Ir_1$, $Ru_{99}Ir_1$-350, $Ru_{95}Ir_5$, $Ru_{95}Ir_5$-350, $Ru_{90}Ir_{10}$, and $Ru_{90}Ir_{10}$-350.

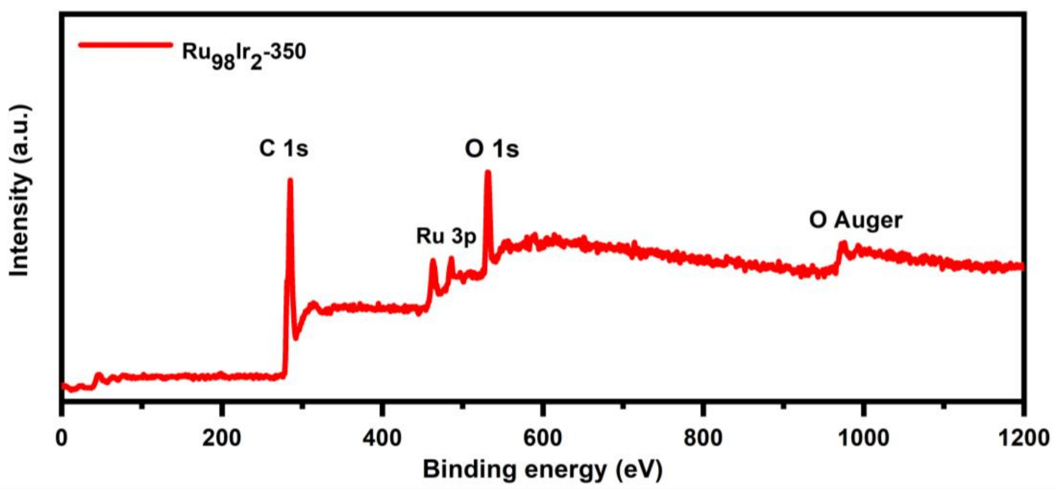

Figure S5. The XPS survey spectrum of $Ru_{98}Ir_2$-350.

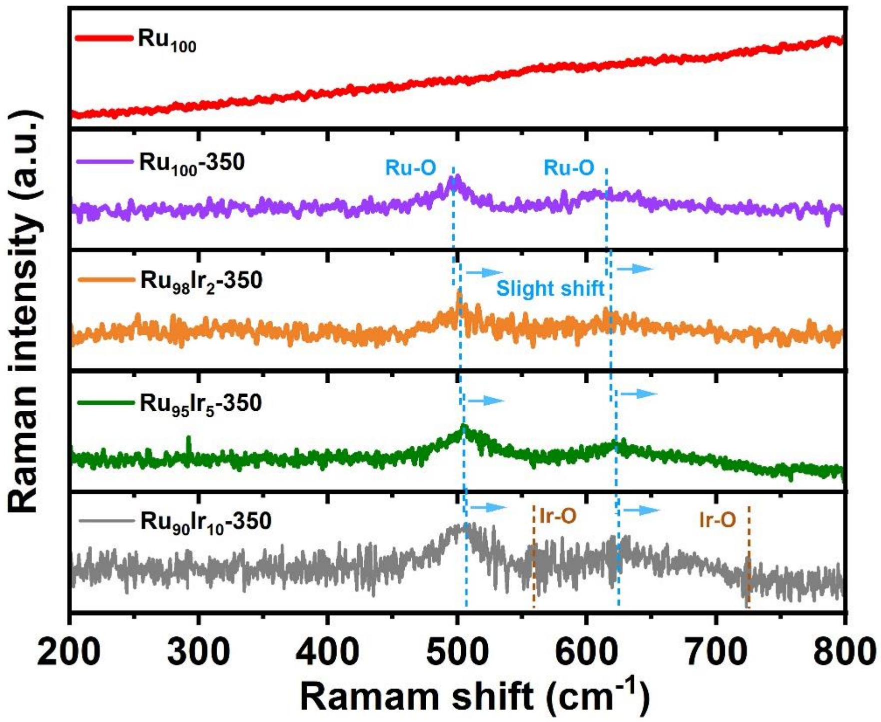

Figure S6. Raman of the as-prepared Ru_{100}, Ru_{100}-350, $Ru_{98}Ir_2$-350, $Ru_{95}Ir_5$-350 and $Ru_{90}Ir_{10}$-350 catalyst.

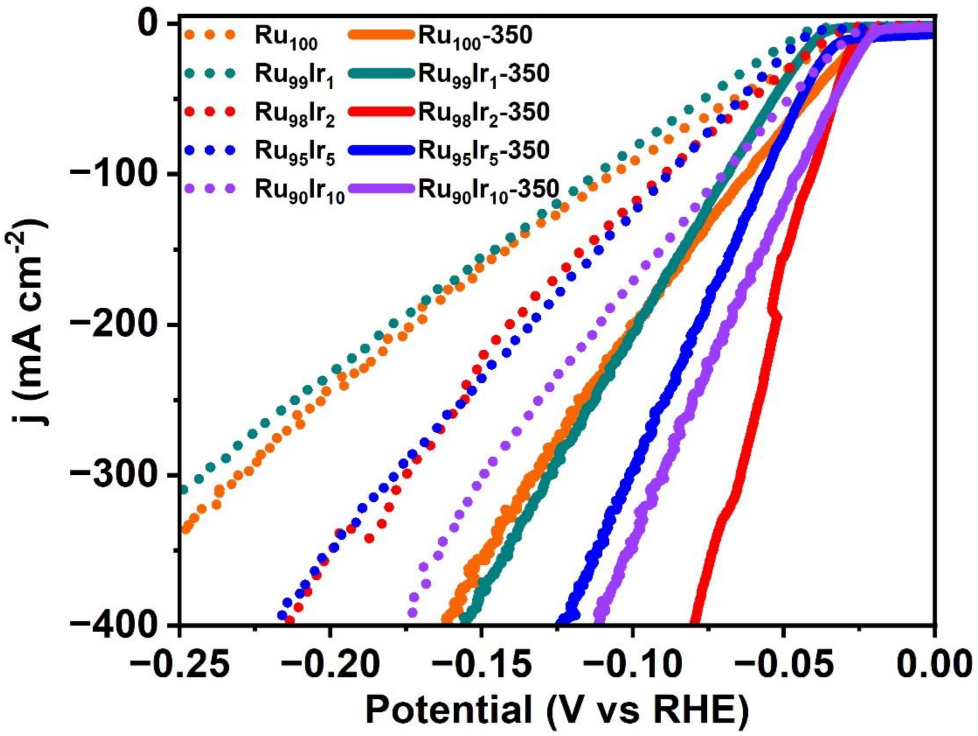

Figure S7. LSV curves of all the as-prepared samples.

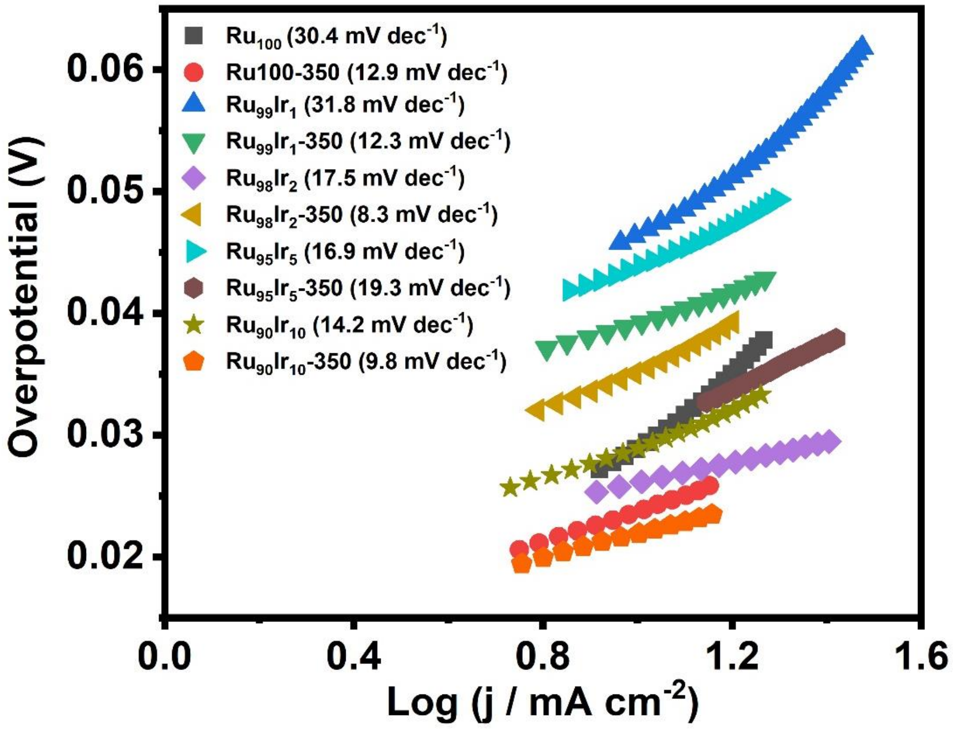

Figure S8. Tafel plots of all the as-prepared samples.

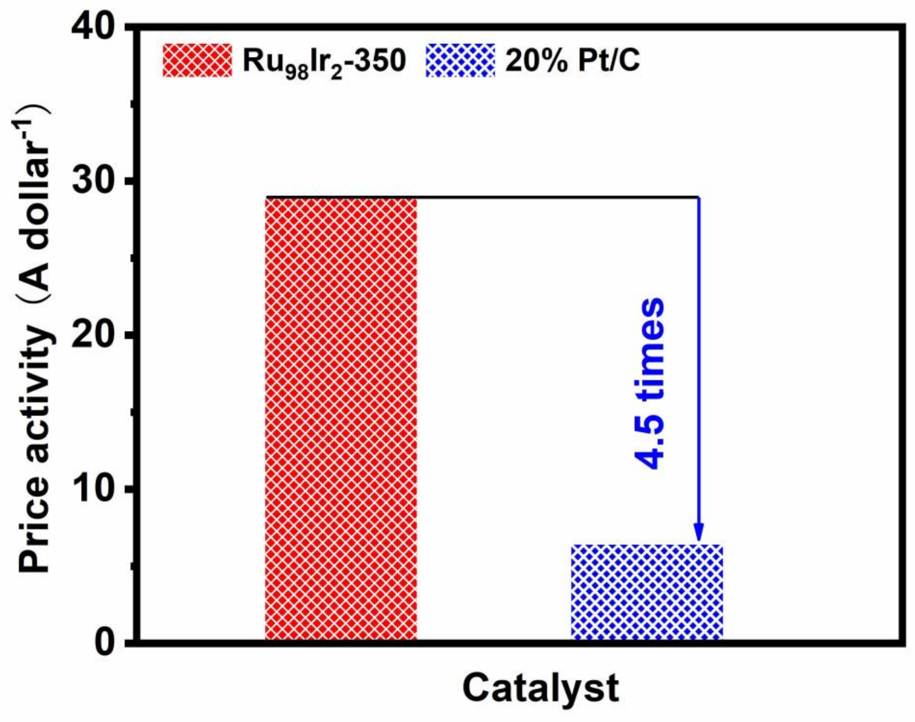

Figure S9. Price activity of samples at an overpotential of 100 mV.

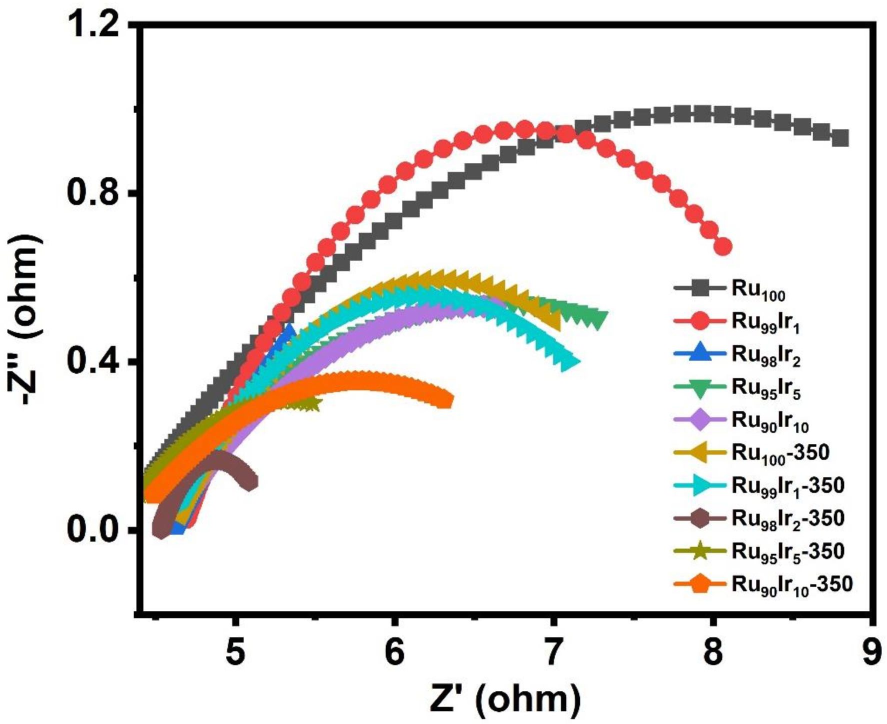

Figure S10. EIS Nyquist plots of all the as-prepared samples.

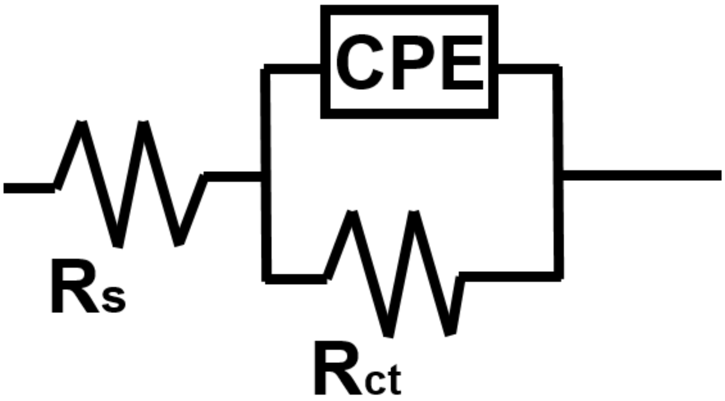

Figure S11. The equivalent circuit model used for simulating the Nyquist plots.

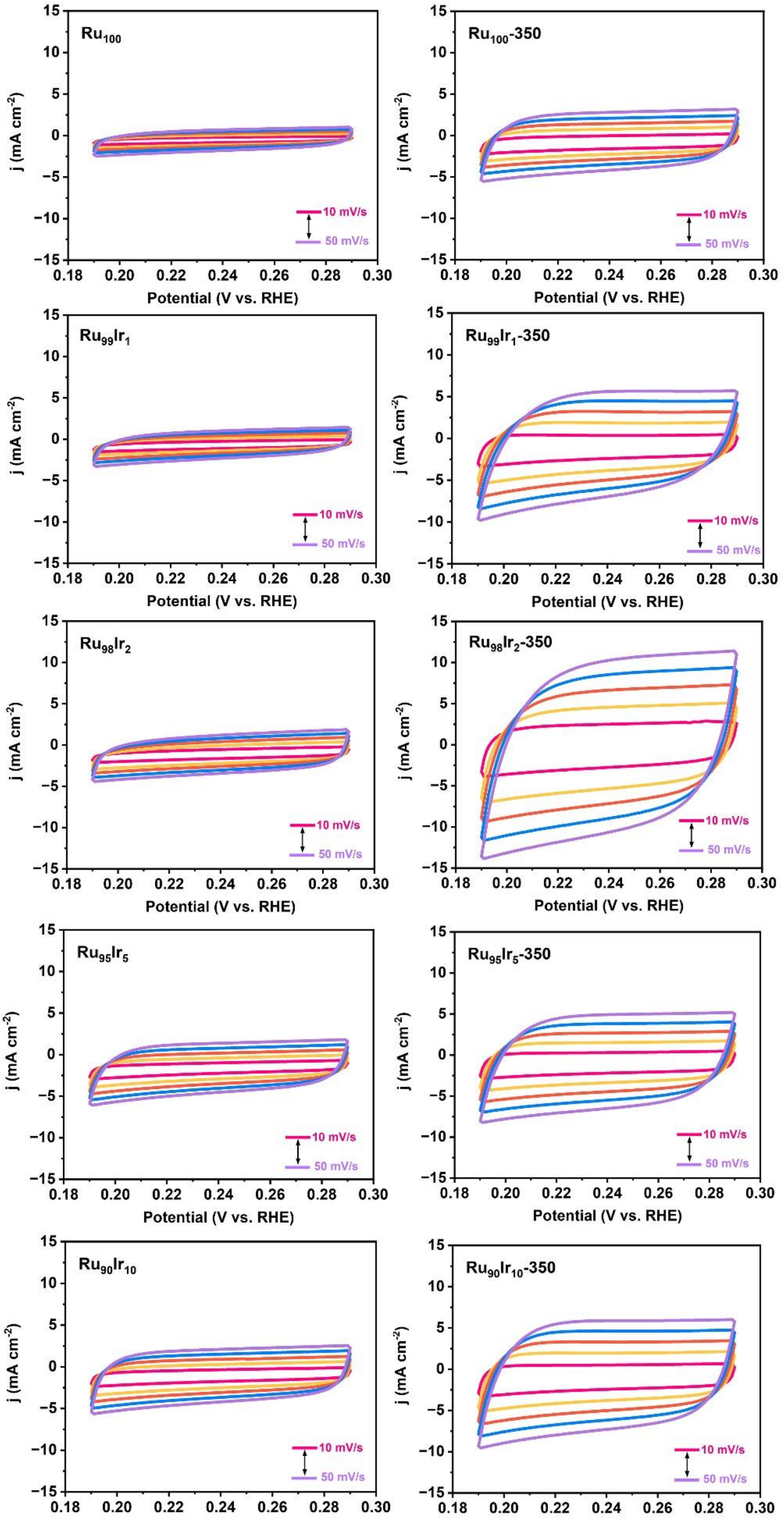

Figure S12. CV curves at different scan rates of varied samples.

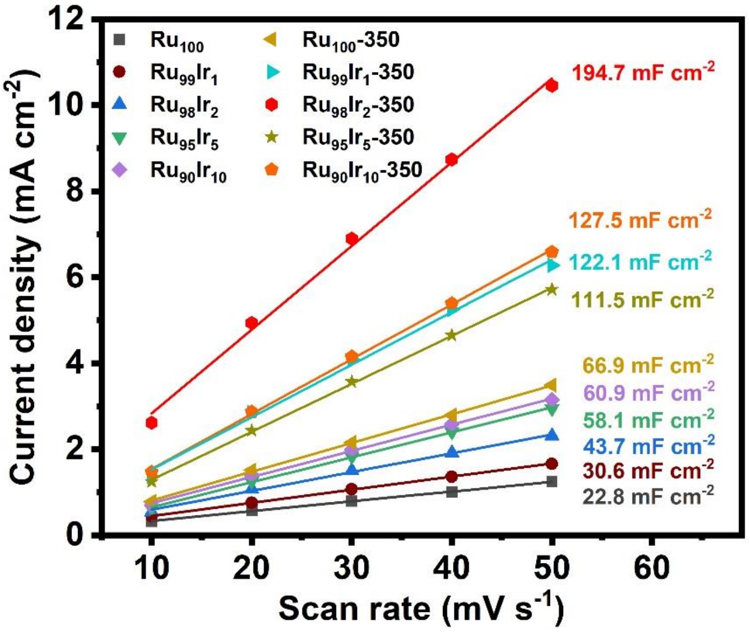

Figure S13. The C_{dl} calculated from capacitive current density vs scan rate of varied samples.

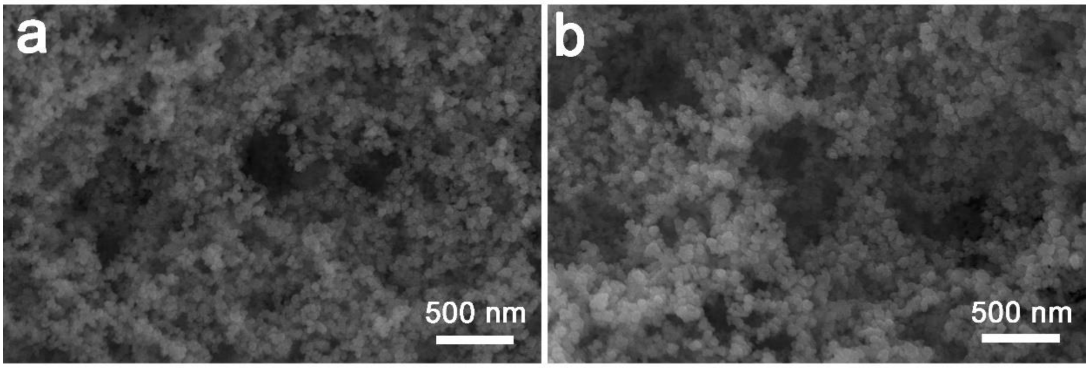

Figure S14. SEM images of (a) $Ru_{98}Ir_2$-200 and (b) $Ru_{98}Ir_2$-500.

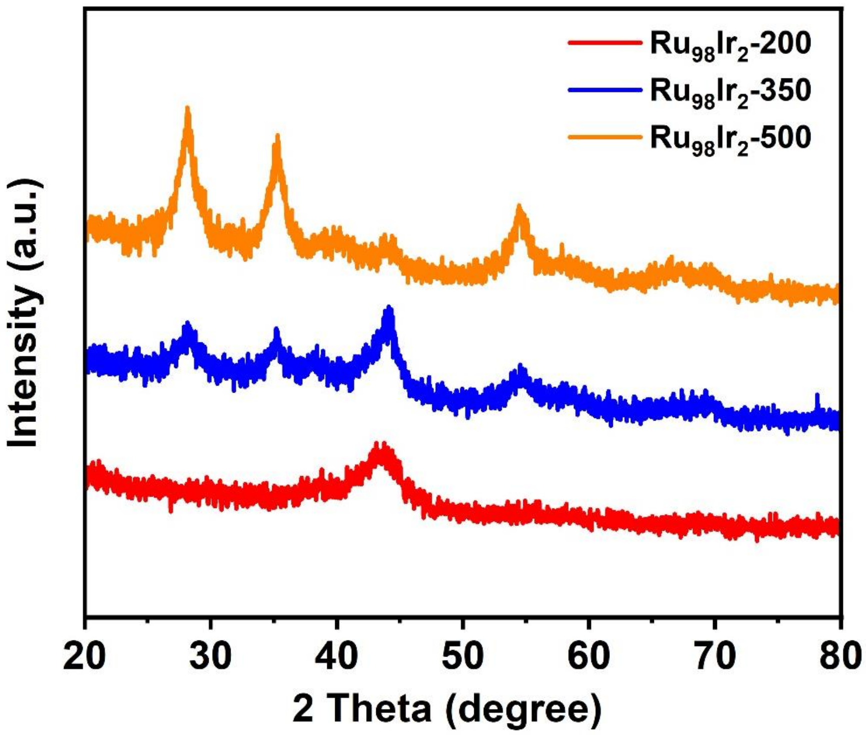

Figure S15. XRD patterns of (a) $Ru_{98}Ir_2$-200 and (b) $Ru_{98}Ir_2$-500.

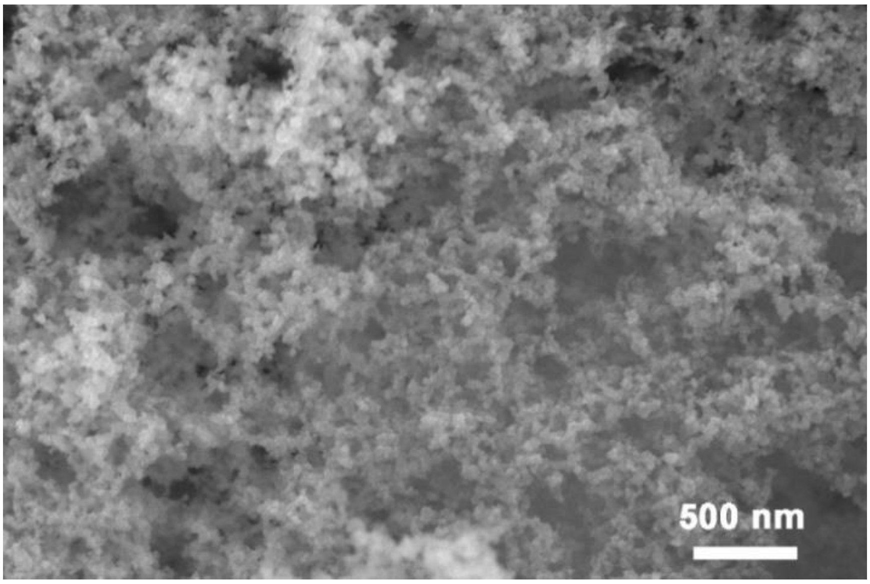

Figure S16. SEM image of the post-HER $Ru_{98}Ir_2$-350 sample.

Table S1. Comparison of the HER performance for $Ru_{98}Ir_2$-350 catalyst with other electrocatalysts in alkaline solution.

Catalyst	Electrolyte	η_{1000} (mV)	Tafel slope (mV dec^{-1})	Reference
$Ru_{98}Ir_2$-350	1 M KOH	121	8.3	This work
Sr_2RuO_4	1 M KOH	278	22	[1]
Ni_3Sn_2-$NiSnO_x$	1 M KOH	165	68.8	[2]
Ru_2@FeCo-LDH/NF	1 M KOH	117	25	[3]
MoS_2/Mo_2C	1 M KOH	220	43	[4]
Mo_2C/MoC/CNT	1 M KOH	233	42	[5]
Ni-MoN	1 M KOH	136	35.5	[6]
Ni/MoO_2 @CN	1 M KOH	267	45	[7]
$Ni(OH)_x/Ni_3S_2$/NF	1 M KOH	238	67	[8]
CoRu-LDH/PANI	1 M KOH	275	71	[9]
Pt/TiO_2/$Ni(OH)_2$	1 M KOH	145	38.9	[10]
Pt/D-NiFe LDH	1 M KOH	217	43	[11]
$Pd_4S/Pd_3P_{0.95}$	1 M KOH	486	67.5	[12]
Fe-PtNiPO-1	1 M KOH	193	35	[13]
Ru-CoO_x/NF	1 M KOH	252	28	[14]

Table S2. Summary for ECSA values of as-prepared catalysts.

Catalyst	ECSA (cm^2)
Ru_{100}	112
$Ru_{99}Ir_1$	150
$Ru_{98}Ir_2$	214
$Ru_{95}Ir_5$	285
$Ru_{90}Ir_{10}$	298
Ru_{100}-350	328
$Ru_{99}Ir_1$-350	598
$Ru_{98}Ir_2$-350	954
$Ru_{95}Ir_5$-350	546
$Ru_{90}Ir_{10}$-350	625

Table S3. Comparison of the current density and time of the $Ru_{98}Ir_2$-350 sample with literature reported HER electrocatalysts.

Catalyst	Electrolyte	Current density (mA cm^{-2})	Stable time (h)	Reference
$Ru_{98}Ir_2$-350	1 M KOH	1000	1500	This work
Ru-W/WO_2-800	1 M KOH	250	500	[15]
Ni_3Sn_2-$NiSnO_x$	1 M KOH	500	500	[2]
Ru_2@FeCo-LDH/NF	1 M KOH	1000	1000	[3]
MoS_2/Mo_2C	1 M KOH	200	24	[4]
Ru/TiN-300	1 M KOH	10	24	[16]
Ni-MoN	1 M KOH	500	200	[6]
Ru@g-CN_x	1 M KOH	10	45	[17]
$Ni(OH)_x$/Ni_3S_2/NF	1 M KOH	320	1000	[8]
Ru-OC_{60}-300/KB	1 M KOH	10	50	[18]
Ru-3/FN	1 M KOH	10	100	[19]
2DPC-RuMo	1 M KOH	10	120	[20]
$CoRu_{0.5}$/CQDs	1 M KOH	20	100	[21]
Ru/RuO_2 SNSs	1 M KOH	25	50	[22]
Ru-MnFeP/NF	1 M KOH	50	50	[23]
2.20wt% Ru SAs-Ni_2P	1 M KOH	100	20	[24]
Ru_1/D-NiFe LDH	1 M KOH	100	50	[25]
Ru/TiO_2-V	6 M KOH	300	200	[26]

Reference

[1] Y. Zhang, K. E. Arpino, Q. Yang, N. Kikugawa, D. A. Sokolov, C. W. Hicks, J. Liu, C. Felser, G. Li, *Nat. Commun.* **2022**, *13*, 7784.

[2] X. Wang, G. Long, B. Liu, Z. Li, W. Gao, P. Zhang, H. Zhang, X. Zhou, R. Duan, W. Hu, C. Li, *Angew. Chem. Int. Ed.* **2023**, e202301562.

[3] X. Mu, X. Gu, S. Dai, J. Chen, Y. Cui, Q. Chen, M. Yu, C. Chen, S. Liu, S. Mu, *Energy Environ. Sci.* **2022**, *15*, 4048.

[4] Y. Luo, L. Tang, U. Khan, Q. Yu, H. M. Cheng, X. Zou, B. Liu, *Nat. Commun.* **2019**, *10*, 269.

[5] C. Li, Z. Wang, M. Liu, E. Wang, B. Wang, L. Xu, K. Jiang, S. Fan, Y. Sun, J. Li, K. Liu, *Nat. Commun.* **2022**, *13*, 3338.

[6] L. Wu, F. Zhang, S. Song, M. Ning, Q. Zhu, J. Zhou, G. Gao, Z. Chen, Q. Zhou, X. Xing, T. Tong, Y. Yao, J. Bao, L. Yu, S. Chen, Z. Ren, *Adv. Mater.* **2022**, *34*, e2201774.

[7] G. Qian, J. Chen, T. Yu, J. Liu, L. Luo, S. Yin, *Nanomicro Lett.* **2021**, *14*, 20.

[8] W. He, R. Zhang, D. Cao, Y. Li, J. Zhang, Q. Hao, H. Liu, J. Zhao, H. L. Xin, *Small* **2023**, *19*, e2205719.

[9] J. Chen, X. Luo, H. Zhang, X. Liang, K. Xiao, T. Ouyang, M. Dan, Z.-Q. Liu, *Electrochim. Acta* **2023**, *439*.

[10] A. Kong, M. Peng, M. Liu, Y. Lv, H. Zhang, Y. Gao, J. Liu, Y. Fu, W. Li, J. Zhang, *Appl. Catal. B-Environ.* **2022**, *316*.

[11] Z. Wu, Z. Chen, K. Xu, B. Li, Z. Li, G. Xu, W. Xiao, T. Ma, Y. Fu, L. Wang, *Renew. Energ.* **2023**, *210*, 196.

[12] G. Zhang, A. Wang, L. Niu, W. Gao, W. Hu, Z. Liu, R. Wang, J. Chen, *Adv. Energy Mater.* **2022**, *12*,

[13] K. Feng, J. Xu, Y. Chen, S. Li, Z. Kang, J. Zhong, *Adv. Sci.* **2022**, *9*,

[14] D. Wu, D. Chen, J. Zhu, S. Mu, *Small* **2021**, *17*,

[15] W. Ma, X. Yang, D. Li, R. Xu, L. Nie, B. Zhang, Y. Wang, S. Wang, G. Wang, J. Diao, L. Zheng, J. Bai, K. Leng, X. Li, Y. Qu, *Adv. Sci.* **2023**.

[16] X. Wang, X. Yang, G. Pei, J. Yang, J. Liu, F. Zhao, F. Jin, W. Jiang, H. Ben, L.

Zhang, *Carbon Energy* **2023**.

[17] T. Y. Gao, K. S. Kumar, Z. Yan, M. Marinova, M. Trentesaux, M. A. Amin, S. Szunerits, Y. Zhou, V. Martin-Diaconescu, S. Paul, R. Boukherroub, V. Ordomsky, *J. Mater. Chem. A* **2023**.

[18] Y. Li, T. Xu, Q. Huang, L. Zhu, Y. Yan, P. Peng, F.-F. Li, *ACS Catal.* **2023**, *13*, 7597.

[19] C. X. Zhang, Y. N. Cui, C. Jiang, Y. X. Li, Z. S. Meng, C. Wang, Z. Y. Du, S. S. Yu, H. W. Tian, W. T. Zheng, *Small* **2023**.

[20] K. Tu, D. Tranca, F. Rodriguez-Hernandez, K. Jiang, S. Huang, Q. Zheng, M. X. Chen, C. Lu, Y. Su, Z. Chen, H. Mao, C. Yang, J. Jiang, H. W. Liang, X. Zhuang, *Adv. Mater.* **2020**, *32*, e2005433.

[21] W. Li, Y. Zhao, Y. Liu, M. Sun, G. I. N. Waterhouse, B. Huang, K. Zhang, T. Zhang, S. Lu, *Angew. Chem. Int. Ed.* **2021**, *60*, 3290.

[22] J. Zhang, G. Ren, D. Li, Q. Kong, Z. Hu, Y. Xu, S. Wang, L. Wang, M. Cao, X. Huang, *Sci. Bull.* **2022**, *67*, 2103.

[23] D. Chen, Z. H. Pu, R. H. Lu, P. X. Ji, P. Y. Wang, J. W. Zhu, C. Lin, H. W. Li, X. G. Zhou, Z. Y. Hu, F. J. Xia, J. S. Wu, S. C. Mu, *Adv. Energy Mater.* **2020**, *10*.

[24] K. Wu, K. Sun, S. Liu, W.-C. Cheong, Z. Chen, C. Zhang, Y. Pan, Y. Cheng, Z. Zhuang, X. Wei, Y. Wang, L. Zheng, Q. Zhang, D. Wang, Q. Peng, C. Chen, Y. Li, *Nano Energy* **2021**, *80*.

[25] P. Zhai, M. Xia, Y. Wu, G. Zhang, J. Gao, B. Zhang, S. Cao, Y. Zhang, Z. Li, Z. Fan, C. Wang, X. Zhang, J. T. Miller, L. Sun, J. Hou, *Nat. Commun.* **2021**, *12*, 4587.

[26] Z. Z. Wei, Z. J. Zhao, J. Wang, Q. Zhou, C. X. Zhao, Z. H. Yao, J. G. Wang, *J. Mater. Chem. A* **2021**, *9*, 10160.
